# Impact of hyperkalemia on hospitalization days in advanced chronic kidney disease patients with Type-2 diabetes mellitus: A prospective study

**DOI:** 10.12669/pjms.39.3.6874

**Published:** 2023

**Authors:** Xiaodong Li, Xinyue Li, Baoxin Li, Yancong Guo

**Affiliations:** 1 Xiaodong Li Department of Nephrology, Baoding No. 1, Central Hospital of Hebei Medical University, Hebei, Baoding 071000, China; 2 Xinyue Li Department of Cardiology, Baoding No. 1, Central Hospital of Hebei Medical University, Hebei, Baoding 071000, China; 3 Baoxin Li Department of Endocrine, Baoding No. 1, Central Hospital of Hebei Medical University, Hebei, Baoding 071000, China; 4 Yancong Guo Department of Nephrology, Baoding No. 1, Central Hospital of Hebei Medical University, Hebei, Baoding 071000, China

**Keywords:** Chronic kidney disease, Diabetes mellitus, Hyperkalemia, Hospitalization days

## Abstract

**Objective:**

This study aimed to explore the impact of hyperkalemia at admission on hospitalization days (HDs) among advanced chronic kidney disease patients (CKD) with type two diabetes mellitus (T2DM) in China.

**Methods:**

A total of 270 CKD patients with T2DM were prospectively selected from January 1, 2020 to December 31, 2021. These patients were divided into Group-A (n = 150, serum potassium ≤ 5.5 mmol/L) and B (n = 120, serum potassium > 5.5 mmol/L). The comparison method between the two groups was taken. Linear correlation analysis was performed using the Spearman correlation method, and multivariate analysis was tested using linear regression.

**Results:**

The study found statistically significant result between the two groups (Group-A vs Group-B): HDs (7.4 (5.3-11.2) vs 12.1 (8.2-16.5), p < 0.001), renin-angiotensin-aldosterone system inhibitors (RAASIs) (36.2% vs 55.8%, p = 0.014), systolic blood pressure (148.35 ± 19.51 vs 162.26 ± 21.31, p < 0.05), estimated glomerular filtration (eGFR) (20.35) (18.31-25.26) vs13.4 (12.50-18.50), p < 0.001, N-terminal pro-B type natriuretic peptide (NT-proBNP) (2245.42 ± 61.09 vs 3163.39 ± 85.15,p < 0.001), and Hb (88.45 ± 12.35 vs 72.26 ± 14.2, p = 0.023). Correlation analysis showed that HDs were positively correlated with age, serum potassium, systolic blood pressure, and NT-proBNP, while negatively with eGFR and Hb. After adjusting for relevant confounding variables, the multivariable linear regression analysis showed that hyperkalemia was an independent risk factor for HDs.

**Conclusions:**

Hyperkalemia could be an independent risk factor increasing HDs of advanced CKD patients with T2DM.

## INTRODUCTION

The prevalence of chronic kidney disease (CKD) has increased recently, mostly accompanied by diabetes, hypertension, and coronary heart disease. Although there was significant variation in overall and advanced CKD prevalence, about 434.3 million adults have CKD in Asia.^1^ It may eventually develop into end-stage renal disease, requiring renal replacement therapy. Particularly, CKD caused by diabetes has become a global health problem.^2^,^3^ The prevalence of hyperkalemia is increasing and CKD is one of the multitude of risk factors for hyperkalemia due to the decline of estimated glomerular filtration rate (eGFR). However, there is still a lack of exact epidemiological data for prevalence of hyperkalemia in CKD patients, which is mainly limited to some local single-center studies. Hyperkalemia is a common yet life-threatening electrolyte disorder in patients with CKD, heart failure, diabetes, or hypertension. Hyperkalemia is reported in 2.6% to 7% general population, while the prevalence in patients with CKD can reach up to 73%, especially in patients with advanced CKD or dialysis.^4^-^6^ Diabetic patients, especially those with CKD, are more susceptible to present with hyperkalemia due to renal disease progression.^7^

Hyperkalemia is one of the prevalent reasons for hospitalization among CKD patients. Some observational studies have described the relationship between hyperkalemia and excessive mortality,^8^ however, there is lack of research on the relationship between hyperkalemia and the length of hospitalization of advanced CKD patients with type two diabetes mellitus (T2DM). As a result, this study aimed to explore the impact of hyperkalemia at admission on hospitalization days (HDS) in advanced CKD patients with T2DM in China, to determine how hyperkalemia would affect financial burdens of hospital, provide a theoretical basis and recommendations for the daily and inpatient management of such patients.

## METHODS

A total of 270 CKD (stage IV and V) patients with T2DM hospitalized at Baoding No. 1 Central Hospital from January 1, 2020 to December 31, 2021 with complete clinical data from Hebei met the diagnostic criteria of T2DM.^9^ According to the criteria of kidney disease improving global outcomes (KDIGO),^10^ CKD staging was performed using creatinine based estimated global filtration rate (eGFR). The eGFR calculation adopts the modification of diet in renal disease (MDRD) equation.^11^

### Inclusion criteria:

- Age ≥ 18 years old;
- CKD stage was stage IV or V.
- Serum potassium was initially checked at the hospital. Group A (non-hyperkalemia group) was taken as serum potassium ≤ 5.5 mmol/L and group B (hyperkalemia group) was taken as serum potassium > 5.5 mmol/L.

### Exclusion criteria:

- Diabetes ketoacidosis, hyperosmolar nonketotic coma, severe infection, liver dysfunction, acute coronary syndrome, rheumatic diseases, and other systemic diseases;
- Pregnant, lactating women or women of childbearing age who intended to conceive;
- Renal artery stenosis;
- Previous history of kidney transplantation, hemodialysis, and peritoneal dialysis;
- Previous decompensation of liver cirrhosis or history of hepatic encephalopathy;
- Any systematic history of malignant tumor disease in the past five years;
- Any existing life-threatening condition or expected survival of less than a year;
- History or evidence of drug or alcohol abuse in the past one year;
- After admission, transferred to the department, discharge automatically, transferred to dialysis or kidney transplantation;
- Combined with acute renal injury.

**Fig.1:**
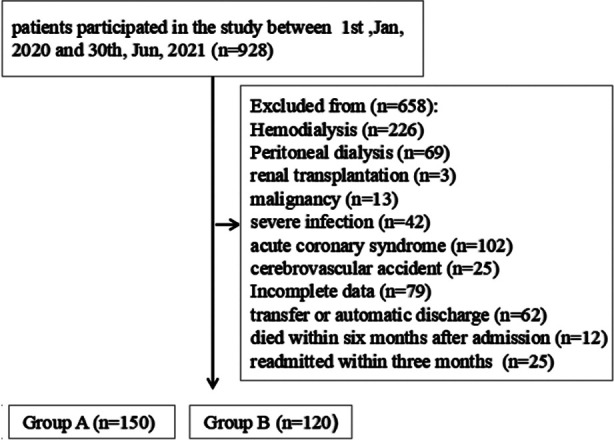
Patients selection flowing chart.

This is a prospective study and analysis. The 270 patients who met the above selection criteria were divided into two groups, including 124 males and 146 females, with 150 patients in the non-hyperkalemia group (Group-A) and 120 patients in the hyperkalemia group (Group-B). The specific selection process of patients. is shown in Fig.1.

### Research Methods:

The general clinical data of patients including age, sex, 24 hours urinary output, body mass index (BMI), diabetic duration, days in hospital, systemic blood pressure (SBP), diastolic blood pressure (DBP), primary disease, and main treatment at admission were collected during hospitalization. The main laboratory examinations included N-terminal pro-B type natural peptide (NT-proBNP), platelet (PLT), hemoglobin (Hb), hemoglobin A1c (HbA1c), albumin (ALB), low density lipoprotein-cholesterol (LDL-C), high density lipoprotein (HDL), total cholesterol (TC), triglyceride (TG), C-reactive protein (CRP), 24 hours urinary protein (24-h Upro), and serum uric acid (SUA).

### Statistical Analysis:

The data was analyzed using SPSS 22.0 statistical software, and PRISM 8.0 was used as a statistical chart. The normality test was performed first for numerical variables. If both groups met the normality with equal variance between them, the mean ± standard deviation was used for statistical description, and the independent sample t-test was used for the comparison between the two groups. Otherwise, the median (upper quartile ~ lower quartile) was utilized for statistical description, and the nonparametric test of independent samples was used for the comparison between the two groups. For categorical variables, the number of cases (%) was used to describe, and the comparison between the two groups adopted χ^2^ inspection. The length of stay was taken as the dependent variable and linear correlation and regression analysis were used. Regression analysis fully corrected various confounding factors. The difference was statistically significant (p < 0.05).

### Ethical Approval:

The studies involving human participants were reviewed and approved by Ethics Committee of Baoding No. 1 Central Hospital. Reference number: 2020-079. The patients provided their written informed consent to participate in this study.

## RESULTS

**Fig.2:**
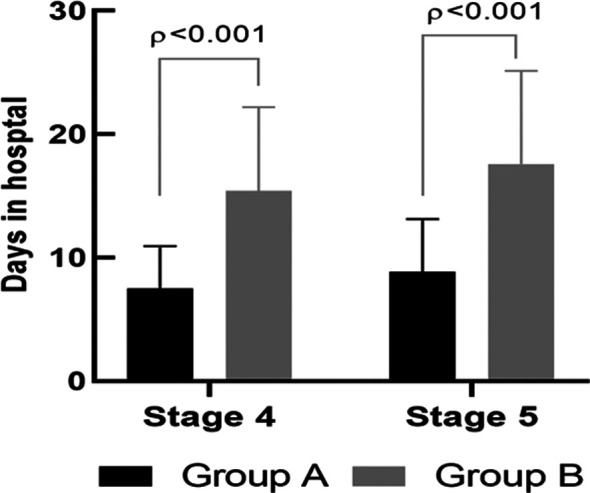
Comparison of inpatient days between the two groups, stratified by CKD stage.

A total of 928 patients were selected, of which 658 patients were excluded and 270 patients were included in this prospective study. Further, 120 patients were categorized in group B (hyperkalemia), accounting for 44.4% of the total inclusion number. Compared to group A, Group B had lower eGFR and Hb, but higher HDs, renin-angiotensin-aldosterone system inhibitors (RAASIs), systolic blood pressure, and NT-proBNP (p < 0.05) (Table-I and II). Compared with the hospitalization time of patients in CKD (stage IV and V), the HDs in the hyperkalemia group were significantly increased (p < 0.001) (Fig.2).

**Table-I:** Comparison of demographic and clinical data between the two groups.

Characteristics	Group A (n=150)	Group B (n=120)	P-value
Gender (male) n (%)	70 (46.23%)	54 (45.98%)	0.582
Age (years)	48.55±10.14	50.12±9.86	0.466
Diabetic duration (years)	8.5( 6.2-15.4)	9.0 (6.8-16.3)	0.276
Days in hospital (days)	7.4(5.3-11.2)	12.1(8.2-16.5)	<0.001
Coronary artery disease (%)	38.5	43.1	0.573
Hear failure (%)	28.4	33.1	0.421
Stroke (%)	15.8	18.4	0.309
Peripheral arterial disease (%)	12.4	15.1	0.247
RAASIs (%)	36.2	55.8	0.014
Potassium-sparing diuretics (%)	15.6	18.3	0.143
Loop diuretic (%)	45.6	48.9	0.425
Insulin (%)	85.6	87.3	0.182
Beta -blocker (%)	32.1	29.8	0.217
Beta-2 agonists (%)	6.3	5.9	0.173
BMI (kg/m²)	26.86±6.25	25.48±5.56	0.213
SBP (mmHg)	148.35±19.51	162.26±21.31	<0.05
DSP (mmHg)	88.65±11.12	81.52±10.96	0.307
24h urinary output (mL)	954.1±125.7	824.2±156.3	0.212

***Abbreviations:*** RAASIs, renin-angiotensin-aldosterone system inhibitors; BMI, body mass index; SBP, systolic blood pressure; DBP, diastolic blood pressure.

**Table-II:** Comparison of laboratory data between the two groups.

Characteristics	Group A (n=150)	Group B (n=120)	P-value
eGFR ( mL/min/1.73m^2^)	20.35 (18.31–25.26)	13.4 (12.50–18.50)	<0.001
Stage 4 CKD (%)	55.8%	51.7	0.217
Stage 5 CKD (%)	44.2%	48.3	0.324
NT-proBNP(pg/mL)	2245.42±61.09	3163.39±85.15	<0.001
Hb (g/L)	88.45±12.35	72.26±14.2	0.023
PLT (×10/L)	213.45±28.36	199.82±32.49	0.203
LDL-C (mmol/L)	4.54±1.65	4.28±1.45	0.412
HDL (mmol/L)	0.76±0.23	0.71±0.29	0.243
TC (mmol/L)	7.25±2.19	7.48±2.47	0.241
TG (mmol/L)	4.36±1.25	4.58±1.37	0.181
HbA1c (%)	7.16±0.4	6.86±0.38	0.075
24hUpro(g)	3.35±0.19	2.98±0.26	0.126
CRP (mg/L)	11.24±1.39	9.98±1.13	0.059
ALB (g/L)	32.25±5.3	30.79±4.2	0.186
SUA(umol/L )	418.45±53.27	436.65±60.53	0.125

***Abbreviations:*** eGFR, estimated glomerular filtration rate, CKD, chronic kidney disease; NT-proBNP, N-terminal pro-B type natriuretic peptide, Hb, hemoglobin; PLT, platelet, LDL-C, low density lipoprotein-cholesterol; HDL, high density lipoprotein; TC, total cholesterol; TG, triglyceride; HbA1c, Hemoglobin A1c, 24hUpro, 24 hours urinary protein, CRP, c-reactive protein, ALB, albumin; SUA, serum uric acid.

**Fig.3:**
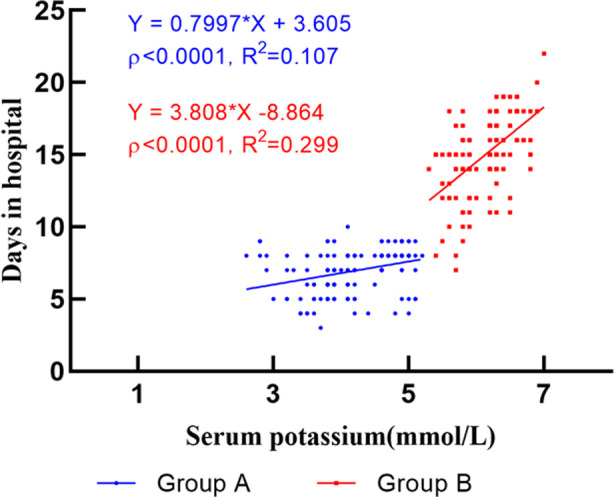
Linear correlation analysis of inpatient days and serum potassium between the two groups.

The total HDs of patients was positively correlated with age, potassium, SBP, and NT-proBNP, and negatively correlated with eGFR and Hb (p < 0.05) (Table-III and Fig.3). Simple linear regression analysis showed HDs were significantly associated with serum potassium, eGFR, NT-proBNP, and Hb (model one). After extensively adjusting for potential confounders (model two, model three, and model four), multiple linear regression analysis showed that hyperkalemia (model four) was still an independently risk factor for HDs (Table-IV).

**Table-III:** Correlation analysis of all patients’ inpatient days (x) and each statistically significant variable (Y).

Variables	Days in hospital(Y)					

	Age (X1)	eGFR(X2)	Hb(X3)	Serum potassium (X4)	SBP(X5)	NT-proBNP (X6)
r-value	0.253	-0.686	-0.543	0.743	0.483	0.153
P-value	<0.05	<0.001	0.023	<0.001	<0.05	0.045

***Abbreviations:*** eGFR, estimated glomerular filtration rate, Hb, hemoglobin, SBP, systolic blood pressure, NT-proBNP, N-terminal pro-B type natriuretic peptide.

**Table-IV:** Independency of hospitalization days of age, eGFR, NT-proBNP, Serum potassium, Hb, SBP.

Variable	Hospitalization days per 1-SD increase							

	Model 1		Model 2		Model 3		Model 4	

	β (95% CI)	P-value	β (95% CI)	P-value	β (95% CI)	P-value	β (95% CI)	P-value
Age	0.24(-0.11/1.12)	0.125						
eGFR	-2.23(-4.52/-1.14)	<0.001	-2.01(-3.45/-1.22)	<0.001	-1.85(-3.37/-1.06)	<0.01	-1.67(-2.82/-1.14)	<0.05
NT-pro BNP	1.14(0.75/2.36)	0.025	0.25(-1.23/2.19)	0.183	1.08(-1.53/3.27)	0.671	0.82(-0.84/2.07)	0.183
Serum potassium	2.35(0.23/3.21)	<0.001	1.48 (1.21/2.23)	<0.05	1.85 (0.74/2.08)	0.014	1.47 (0.81/2.56)	0.025
Hb	-2.12(-4.28/-1.09)	0.014	-1.03(-4.05/0.79)	0.246	-0.58(-3.28/0.46)	0.453	-0.87(-3.93/0.49)	0.358
SBP	0.85(-1.21/2.23)	0.386						

***Abbreviations:*** eGFR, estimated glomerular filtration rate, NT-proBNP, N-terminal pro-B type natriuretic peptide, Hb, hemoglobin, SBP, systolic blood pressure, and other abbreviations are same as Table-I and Table-II. Unadjusted (model-1), model-1 adjusting for sex (model-2), model-2 added RAASIs, BMI, DBP, 24h urinary output (model-3), model-3 added LDL-C, HbA1c, 24hUpro, ALB, CRP and UA (model-4).

## DISCUSSION

This study is a prospective clinical analysis and single center research. It was found that from January 1, 2020 to December 31, 2021, 270 CKD (stage IV and V) patients with T2DM were hospitalized, of which 44.4% had hyperkalemia. D’Alessandro et al discovered that the prevalence of hyperkalemia was associated with decreased eGFR, up to 36.5% for eGFR < 20 ml/min,^12^ which is consistent with our results. Compared with non-hyperkalemic patients, hyperkalemic patients had lower eGFR and Hb, higher application rate of RAASIs, higher systolic blood pressure and NT-proBNP, and longer HDs. RAASIs are widely used in patients with CKD, hypertension, and diabetes due to its unique effects of improving cardiac and renal function and reducing urinary protein. However, they may increase the risk of hyperkalemia.^13^ Moreover, our study discovered that the application of RAASIs is related to hyperkalemia, and our study still confirmed that low eGFR was a significant factor related to hyperkalemia.^14^

Hyperkalemia is a common electrolyte disorders in the middle and late stages of CKD, which can be caused by various pathophysiological mechanisms.^15^ CKD is often associated with decreased GFR, tubulointerstitial dysfunction, and metabolic acidosis.^16^ Patients with diabetes, insulin deficiency, hyperglycemia, and hypoproteinemia are susceptible to hyperkalemia, especially with acute heart failure.^17^

The correlation analysis between the HDs and its primary variables in our patients displayed that the HDs were negatively correlated with Hb and eGFR, but positively correlated with age, SBP, and serum potassium. The multivariable linear regression analysis showed that eGFR was an independent protective factor, which could reduce HDs, while hyperkalemia was an independent risk factor, which could significantly increase HDs. Although the findings are consistent with the results of Calabrese et al,^18^ the subjects of our study were advanced CKD patients with T2DM, and more such patients were included. Regression analysis have widely adjusted for the influence of other related confounding factors, hence our result precisely proved that hyperkalemia is an independent risk factor for prolonging the HDs of advance CKD patients with T2DM.

Hougen et al 17 performed a retrospective cohort study using administrative databases in Canada and founded that among 93,667 patients with de novo hyperkalemia, 36% had diabetes mellitus (DM), 28% had CKD, and 21% had heart failure (HF). In this propensity score-matched sample of 177,082 individuals, hyperkalemia was associated with an increased risk for all-cause mortality, cardiovascular events, short-term mortality, hospitalizations and ICU admissions. Another matched cohort research from China found^19^ that Among 1,003 pairs of CKD patients (mean age 67.2 ± 14.3 years), the mean length of stay per admission and number of admissions per patient among the hyperkalemia cohort were significantly higher than the non-hyperkalemia cohort (P < 0.001) in each follow-up month, and those hyperkalemia patients had more annual inpatient admissions (1.9 vs. 0.7) and length of stays (28.6 vs. 8.7), while the number of outpatient visits (36.8 vs. 36.4) were similar. Though this result comes from the retrospective data, the conclusion is completely accordant with ours study.

Therefore, Hyperkalemia in advanced CKD with diabetes is prevalent and burdensome, causing more harm, more complications and higher medical costs, which should be given sufficient attention by medical staff. 20

Renal function is significantly damaged when coupled with the aging of patients and the complexity of various drugs and dialysis treatment makes the mechanism of hyperkalemia more complex.^21^ The decline of renal function is the primary factor for increased serum potassium levels. It is necessary to manage serum potassium according to eGFR because abnormal potassium metabolism will lead to fatal arrhythmia or sudden cardiac death, especially for CKD patients with T2DM using RAASIs, serum potassium should be routinely monitored and early and appropriate interventions should be provided.^22^-^24^

### Limitations:

First, this study is a single-centered clinical analysis, with limited samples due to regional restrictions. Secondly, the design of this study failed to clarify the etiology or inducement of hyperkalemia in patients, such as acid-base imbalance, diet, and other factors, and it did not dynamically monitor the change in serum potassium level. Lastly, we did not include all the treatments for those patients, such as therapeutic fluids, some herbal medicine, sodium polystyrene sulfonate and other potassium lowering drugs that may affect serum potassium, making it difficult to completely control the influence of other confounding factors. As a result, the findings of this study must be integrated with the actual clinical situation, and further prospective multicenter research is required to confirm.

## CONCLUSION

In this study, CKD (stage IV and V) patients with T2DM were hospitalized, and the risk factors affecting HDs were analyzed systematically. Hyperkalemia was found to be an independent risk factor affecting HDs. This finding highlights the urgent need for efficient serum potassium management measures in hospital in order to avoid the acutely life-threatening effects of hyperkalemia. Furthermore, this study provides a theoretical and clinical basis for preventing and treating hyperkalemia in such patients, which will obviously prevent CKD patients with T2DM from the undesirable outcomes and inpatient economic burdens. Routine monitoring, rational drug use, early detection, and early treatment measures are crucial, which can shorten HDs and reduce the hospitalization expenses of such patients.
